# Plasma phosphate and all-cause mortality in individuals with and without type 2 diabetes: the Dutch population-based lifelines cohort study

**DOI:** 10.1186/s12933-022-01499-4

**Published:** 2022-04-27

**Authors:** Amarens van der Vaart, Qingqing Cai, Ilja M. Nolte, André P. J. van Beek, Gerjan Navis, Stephan J. L. Bakker, Peter R. van Dijk, Martin H. de Borst

**Affiliations:** 1 Department of Medicine, Division of Nephrology, University Medical Centre Groningen, University of Groningen, P.O. Box 30.001, 9700 RB Groningen, The Netherlands; 2 Department of Medicine, Division of Endocrinology, University Medical Centre Groningen, University of Groningen, P.O. Box 30.001, 9700 RB Groningen, The Netherlands; 3 National Clinical Research Center for Kidney Disease, State Key Laboratory of Organ Failure Research, Division of Nephrology, Nanfang Hospital, Southern Medical University, Guangzhou, China; 4 Department of Epidemiology, University Medical Center Groningen, University of Groningen, Groningen, The Netherlands

**Keywords:** Phosphate, Type 2 diabetes, All-cause mortality

## Abstract

**Introduction:**

Individuals with type 2 diabetes have a substantially elevated cardiovascular risk. A higher plasma phosphate level promotes vascular calcification, which may adversely affect outcomes in individuals with type 2 diabetes. We hypothesized that the association between plasma phosphate and all-cause mortality is stronger in individuals with type 2 diabetes, compared to those without diabetes.

**Methods:**

We analysed the association between plasma phosphate and all-cause mortality in the Dutch population-based Lifelines cohort and in subgroups with and without type 2 diabetes, using multivariable Cox regression adjusted for potential confounders. Effect modification was tested using multiplicative interaction terms.

**Results:**

We included 57,170 individuals with 9.4 [8.8–10.4] years follow-up. Individuals within the highest phosphate tertile (range 1.00–1.83 mmol/L) were at higher risk of all-cause mortality (fully adjusted HR 1.18 [95% CI 1.02–1.36], p = 0.02), compared with the intermediate tertile (range 0.85–0.99 mmol/L). We found significant effect modification by baseline type 2 diabetes status (p-interaction = 0.003). Within the type 2 diabetes subgroup (N = 1790), individuals within the highest plasma phosphate tertile had an increased mortality risk (HR 1.73 [95% CI 1.10–2.72], p = 0.02 vs intermediate tertile). In individuals without diabetes at baseline (N = 55,380), phosphate was not associated with mortality (HR 1.12 [95% CI 0.96–1.31], p = 0.14). Results were similar after excluding individuals with eGFR < 60 mL/min/1.73 m^2^.

**Discussion:**

High-normal plasma phosphate levels were associated with all-cause mortality in individuals with type 2 diabetes. The association was weaker and non-significant in those without diabetes. Measurement of phosphate levels should be considered in type 2 diabetes; whether lowering phosphate levels can improve health outcomes in diabetes requires further study.

**Supplementary Information:**

The online version contains supplementary material available at 10.1186/s12933-022-01499-4.

## Introduction

The prevalence of type 2 diabetes has doubled over the past 20 years and now affects 9% of the global population [1]. Moreover, the risk of premature mortality in individuals with type 2 diabetes is two to fourfold higher compared to the general population [2]. Cardiovascular disease (CVD) is the leading cause of death in the general population globally (~ 32%), and is an even more common cause of death in type 2 diabetes (~ 50%) [3, 4]. Traditional risk factors explain only ~ 35% of the excessive CVD risk in type 2 diabetes [5]. Therefore, it is crucial to identify novel potentially modifiable pathways that promote the development and progression of (CVD-related) morbidity and mortality in type 2 diabetes.

Individuals with type 2 diabetes are prone to develop progressive calcification and stiffening of the arterial lamina media, increasing the risk of hypertension, cardiovascular events including stroke and myocardial infarction [6–8], and premature mortality [9]. Vascular calcification is regarded as the consequence of a disequilibrium of endogenous and exogenous factors that promote or inhibit calcification [10]. Phosphate is a major calcification promotor, inducing a phenotypic change in vascular smooth muscle cells (VSMCs) towards a bone phenotype (osteochondrogenic differentiation) through phosphate transporter-1 (PiT-1/SLC20A1) [11, 12]. Of note, in vitro studies demonstrated upregulation of PiT-1 expression in VSMCs under high-glucose conditions [13, 14]. Moreover, high phosphate in the context of high glucose led to more pronounced calcification and higher calcium content of cultured VSMCs, compared with normoglycemic conditions. This suggests that hyperglycemia makes VSMCs more susceptible to phosphate-induced vascular calcification. Higher phosphate levels are independently associated with cardiovascular and all-cause mortality in the general population [15]. Yet, whether this suggested increased susceptibility translates into a stronger association with mortality in individuals with diabetes has not been addressed.

Therefore, the main aim of the current study was to study the association between plasma phosphate and all-cause mortality in a large, population-based cohort study, and to explore potential effect modification by baseline type 2 diabetes status.

## Methods

### Study design and participants

The population-based Lifelines cohort study consists of more than 165,000 participants in the Northern region of the Netherlands with longitudinal follow-up. Participants were recruited between 2006 and 2011 upon invitation by their general practitioner (GP), and after inclusion, if possible, children and parents of the participants were also approached. In addition, registration via the Lifelines website was possible for those who did not receive an invitation from their GP. Detailed information about the Lifelines Cohort Study can be found elsewhere [16]. The Lifelines Cohort Study is conducted according to the principles of the Declaration of Helsinki and was approved by the Institutional Review Board of the University Medical Center Groningen (2007/152).

The present study was restricted to individuals with available laboratory data (including plasma phosphate); therefore, those who did not participate in baseline blood sample collection were excluded. Also, all individuals that use prescribed phosphate binders or phosphate supplements and individuals with type 1 diabetes or Fanconi syndrome were excluded.

### Data collection

Information regarding the (socio) demographic characteristics, medical history, alcohol use and drugs habits was collected by self-administered questionnaires. Education level was classified into four categories (low: never been to school or elementary school only or lower vocational or secondary school; middle: intermediate vocational school or intermediate/higher secondary school; high, higher vocational school or university; unknown or no answer). Marriage status was classified into seven categories [married/registered partnership, cohabiting, single, widow/widower, divorced, other, in a serious relationship (not cohabiting)]. Exercise was calculated according to self-reported non-occupational vigorous activity in minutes per week (SQUASH questionnaire, described elsewhere [17]). Authorized technicians measured height, weight and blood pressure. Blood pressure was repeatedly measured, ten times in ten minutes with a Dynamap PRO 100V2 device. The average of the final three measurements was registered [16].

Laboratory tests were conducted once at the time of cohort entry. Plasma sodium, potassium, phosphate and glucose were determined routinely on a Cobas 8000 platform (Roche, Mannheim, Germany), the latter using a hexokinase UV test. HbA1c concentrations were measured on a Tosoh G8 (HPLC, Sysmex Corporation, Norderstedt, Germany). The plasma phosphate normal range for adults was defined as levels between 0.70 and 1.50 mmol/L. Estimated glomerular filtration rate (eGFR) was calculated according to the Chronic Kidney Disease Epidemiology Collaboration equation (CKD-EPI).

We studied the influence of diet quality on plasma phosphate by using the Lifelines Diet Score (LLDS), a food-based diet score that was developed using international literature, as extensively described elsewhere[18]. BMI was categorized as underweight (< 18.5), normal weight (18.5–24.9), overweight (25.0 – 29.9) and obesity (> 30). Use of medication [e.g., antidiabetic drugs, diuretics, anti-thrombotic agents, lipid lowering drugs and vitamin D (both colecalciferol and active vitamin D analogues)] was assessed by ATC codes in a verified database of registered drugs for each participant. Individuals were considered to have type 2 diabetes if they had either: (1) self-reported type 2 diabetes, (2) a non-fasting plasma glucose > 11 mmol/L, (3) a glycated haemoglobin ≥ 48 mmol/mol, or (4) use of blood glucose lowering drugs [19]. A history of cardiovascular disease included all self-reported myocardial infarction, heart failure, atrial fibrillation, heart valve disorders, arrhythmia, aneurysm, stroke, thrombosis, atherosclerosis, narrowing carotid arteries, and a history of coronary artery bypass grafting (CABG). Mortality was determined according to municipal registers.

### Statistical analysis

All statistical analyses were performed with SPSS software, version 23.0 for Windows (IBM, Armonk, NY), and R version 3.4.2 (Vienna, Austria). In all analyses, a two-sided p value < 0.05 was considered statistically significant.

Normality was tested with histograms and probability plots. Normally distributed variables are presented as mean ± SD and skewed variables as median [interquartile range]. Categorical variables are presented as absolute numbers (%). Baseline characteristics were compared between tertiles of plasma phosphate using one-way ANOVA, Kruskal–Wallis test, and chi-square tests as appropriate. We similarly compared baseline characteristics of participants with and without available laboratory data. Homogeneity of variances was tested using Levene’s test. Log-transformation was performed for not normally distributed data when applied for Cox regression.

Multivariate Cox regression models were used to test the association between plasma phosphate levels and mortality. Since we anticipated a non-linear association between plasma phosphate levels and mortality risk, we analysed plasma phosphate according to tertiles in main analyses, and defined the intermediate tertile as reference group [20]. Covariates were selected for multivariable models if significantly association with all-cause mortality upon univariable Cox regression analysis (inclusion if p < 0.20) or if the covariate was considered clinically relevant. Model 1 was a basic model adjusted for age and gender. In Model 2, we additionally adjusted for smoking, body mass index (BMI), glucose, HbA1c, and systolic blood pressure (SBP). Finally, in Model 3, we additionally adjusted for LDL cholesterol, history of CVD, eGFR, (corrected) calcium, and vitamin D suppletion. Nonlinearity was tested using the likelihood ratio test, comparing nested models with linear and cubic spline terms. Effect modification by age, gender, BMI, and eGFR was tested in the fully adjusted model (Model 3), containing both main effect and their cross-product term. Effect modification by type 2 diabetes status was tested by models containing main effect and multiplicative terms of its cross-product term. The association between plasma phosphate and all-cause mortality was visualized with fully adjusted restricted cubic splines using three knots (25th, 50th, and 75th percentile). The median was taken as reference for all spline plots.

We performed sensitivity analyses by repeating identical Cox regression models in subpopulations restricted to individuals with eGFR ≥ 60 at baseline or individuals with phosphate levels within the normal range. Finally, in a third sensitivity analysis, we excluded individuals that had died within the first five years of follow-up.

## Results

### Baseline characteristics

**Fig. 1:**
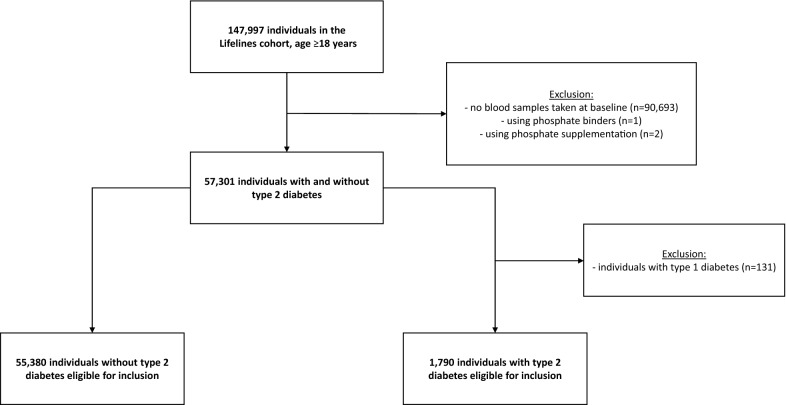
Selection of individuals

A total of 57,170 individuals were included in the present study (Fig. 1). Baseline characteristics according to tertiles of plasma phosphate are presented in Table 1. Median age was 44 [36–51] years and 59% were female. The average plasma phosphate at baseline was 0.91 ± 0.17 mmol/L and eGFR 96 ± 15 mL/min/1.73 m^2^. Participants in the highest plasma phosphate tertile tended to be less frequently male, had lower systolic and diastolic blood pressure, had lower levels of low-density lipoprotein (LDL) cholesterol and triglycerides, higher LLDS (reflecting better diet quality), and higher levels of high-density lipoprotein (HDL) cholesterol. Baseline characteristics were comparable between individuals with and without available laboratory data at baseline (Additional file 1: Table S1).

**Table 1:** Baseline characteristics of participants according to tertiles of plasma phosphate in the Lifelines cohort

	Total (*n* = 57 170)	T1 (*n* = 19 898)	T2 (*n* = 18 355)	T3 (*n* = 18 917)	p for trend
		Plasma phosphate	Plasma phosphate	Plasma phosphate	
		0.24–0.84 mmol/L	0.85–0.99 mmol/L	1.00–1.83 mmol/L	
*Demographics*					
Age (years)	44 [36–51]	45 [38–50]	44 [36–50]	44 [34–51]	** < 0.001**
Gender (male, %)	23,633(41.2)	12,036 (61)	7018 (38)	4510 (24)	** < 0.001**
Smoking					
No smoking (%)	22,951 (43)	8201 (44)	7431 (43)	7274 (41)	** < 0.001**
Former smoker (%)	20,056 (37)	7258 (39)	6299 (37)	6448 (36)	** < 0.001**
Current smoker (%)	11,054 (20)	3380 (18)	3522 (20)	4136 (23)	** < 0.001**
Alcohol use (units/week)	7 [0–14]	14 [0–21]	7 [0–14]	0 [0–14]	** < 0.001**
BMI (kg/m^2^)	26.1 ± 4.3	26.8 ± 4.2	26.1 ± 4.3	25.2 ± 4.3	** < 0.001**
Lifelines Diet Score	24.0 ± 6.0	23.3 ± 5.9	24.1 ± 6.0	24.7 ± 6.2	** < 0.001**
Systolic blood pressure (mmHg)	126 ± 15	129 ± 15	126 ± 15	123 ± 15	** < 0.001**
Diastolic blood pressure(mmHg)	74 ± 9	76 ± 9	74 ± 9	72 ± 9	** < 0.001**
History of cardiovascular disease (%)	1519 (3)	586 (3)	471 (3)	458 (2)	**0.004**
Diabetes (%)	1790 (3)	575 (3)	562 (3)	653 (3)	**0.005**
Other comorbidities (COPD, cancer, dementia, epilepsy, liver cirrhosis) (%)	325 (1)	128 (1)	105 (1)	92 (1)	0.12
Sodium (mmol/L)	142 ± 2	142 ± 2	142 ± 2	142 ± 2	** < 0.001**
Potassium (mmol/L)	3.9 ± 0.3	3.9 ± 0.3	3.9 ± 0.3	3.9 ± 0.3	**0.02**
Calcium (mmol/L)	2.28 ± 0.08	2.27 ± 0.09	2.27 ± 0.08	2.28 ± 0.08	** < 0.001**
eGFR (ml/min/1.73 m^2^)	96 ± 15	95 ± 14	96 ± 15	96 ± 16	** < 0.001**
Phosphate (mmol/L)	0.91 ± 0.17	0.73 ± 0.09	0.92 ± 0.04	1.10 ± 0.09	
Glucose (mmol/L)	5.0 ± 0.9	5.1 ± 0.8	5.0 ± 0.8	4.9 ± 0.8	** < 0.001**
HbA1c (mmol/mol)	38 ± 5	38 ± 5	38 ± 5	38 ± 5	** < 0.001**
HbA1c (%)	5.6 ± 0.7	5.6 ± 0.7	5.6 ± 0.7	5.6 ± 0.7	** < 0.001**
HDL cholesterol (mmol/L)	1.46 ± 0.40	1.37 ± 0.35	1.47 ± 0.38	1.56 ± 0.41	** < 0.001**
LDL cholesterol (mmol/L)	3.19 ± 0.90	3.25 ± 0.87	3.17 ± 0.90	3.15 ± 0.92	** < 0.001**
Triglycerides (mmol/L)	0.99 [0.72–1.42]	1.09 [0.79–1.56]	0.97 [0.71–1.39]	0.91 [0.68 -1.30]	** < 0.001**
Total cholesterol (mmol/L)	5.03 ± 1.00	5.05 ± 0.96	5.00 ± 0.99	5.04 ± 1.03	** < 0.001**
Vitamin D supplement use (%)	127 (0)	34 (0)	41 (0)	52 (0)	0.09
Use of diuretics (%)	2023 (4)	729 (4)	638 (4)	656 (4)	0.50
Use of lipid lowering drugs (%)	3332 (6)	1174 (6)	1021 (6)	1137 (6)	0.16
Use of anti-thrombotic agents (%)	2082 (4)	834 (4)	634 (4)	614 (3)	** < 0.001**

Values are means ± standard deviation, medians (interquartile range) or proportions (%)

P values of < 0.05 were considered as clinical significant and are presented in bold

*BMI* body mass index, *eGFR* estimated glomerular filtration rate, *HbA1c* glycated haemoglobin, *HDL* high-density lipoprotein, *LDL* low-density lipoprotein, *COPD* chronic obstructive pulmonary disease

### Plasma phosphate and all-cause mortality

**Fig. 2:**
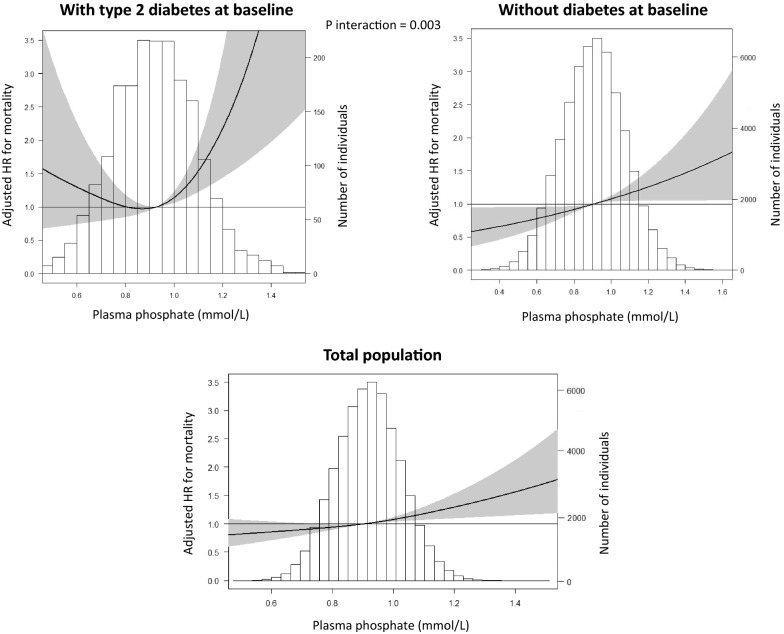
Plasma phosphate and all-cause mortality in the total population and in subgroups of individuals with type 2 diabetes *versus* without diabetes at baseline

During median follow-up for 9.4 [8.8–10.4] years, 1265 individuals (2.2%) died. Characteristics of all individuals that had died are presented in Additional file 1: Table S2. After adjustment for potential confounders, individuals in the highest plasma phosphate tertile (range 1.00–1.83 mmol/L) had a higher mortality risk (fully adjusted hazard ratio 1.18; 95% CI 1.02–1.36), compared with individuals in the intermediate (reference) tertile (range 0.85–0.99 mmol/L) (Table 2). Furthermore, individuals in the lowest tertile had a similar mortality risk compared to those in the intermediate tertile (fully adjusted hazard ratio 0.98; 95% CI 0.85–1.12). As shown in Fig. 2, the association between plasma phosphate and mortality in individuals with type 2 diabetes at baseline was J-shaped, while this association was linear for individuals without type 2 diabetes at baseline.

**Table 2:** Associations between plasma phosphate and all-cause mortality in the full Lifelines cohort

	Tertile 1	Tertile 2	Tertile 3	*p*
	0.24–0.84 mmol/L	0.85–0.98 mmol/L	0.99–1.83 mmol/L	
Person years	189,118	176,525	183,472	
Events	463	375	427	
Crude incident rate per 1000 person-years	2.45	2.12	2.33	
Model 1	0.97 (0.84–1.12)	1.0 (ref)	**1.23 (1.07–1.42)**	**0.01**
Model 2	0.99 (0.86–1.13)	1.0 (ref)	**1.18 (1.02–1.36)**	**0.03**
Model 3	0.99 (0.85–1.13)	1.0 (ref)	**1.17 (1.02–1.35)**	**0.03**
Model 4	0.98 (0.85–1.12)	1.0 (ref)	**1.18 (1.02–1.36)**	**0.02**

Data are presented as hazard ratio (HR) plus 95% CI according to tertiles of plasma phosphorus

P values of < 0.05 were considered as clinical significant and are presented in bold

Model 1: adjusted for age and gender

Model 2: adjusted for Model 1 plus smoking, use of alcohol, BMI (categorical), HbA1c, and SBP

Model 3: adjusted for Model 2 plus LDL, eGFR, (corrected) plasma calcium, use of lipid lowering drugs, use of anti-thrombotic agents, use of anti-diabetic agents, use of diuretics, and use of vitamin D supplementation

Model 4: adjusted for Model 3 plus LLDS, education level, income, marital stage, exercise, and presence of comorbidities (COPD, cancer, dementia, epilepsy, liver cirrhosis, history of CVD)

*BMI* body mass index, *HbA1c* glycated haemoglobin, *SBP* systolic blood pressure, *LDL* low-density lipoprotein, *CVD* cardiovascular disease, *eGFR* estimated glomerular filtration rate, *LLDS* Lifelines Diet score, *COPD* chronic obstructive pulmonary disease, *CVD* cardiovascular disease

We subsequently explored potential effect modification by baseline type 2 diabetes status, age, gender, smoking, and eGFR for the association between plasma phosphate and all-cause mortality. We found relatively strong effect modification by baseline type 2 diabetes status (p-interaction = 0.003), and weaker effect modification by age (p-interaction = 0.01) and active smoking (p-interaction = 0.05). All other variables were non-significant (p-interaction > 0.10). Characteristics of the subpopulation with type 2 diabetes at baseline are presented in Additional file 1: Table S3.

Table 3 shows the results from multivariable Cox regression analyses according to type 2 diabetes status at baseline. For both populations (with and without type 2 diabetes at baseline), we used identical cut-off values to define three plasma phosphate groups. In individuals with type 2 diabetes at baseline, those with the highest plasma phosphate levels had a higher risk of all-cause mortality, compared with the intermediate group, whereas this association was non-significant for individuals without type 2 diabetes at baseline.

**Table 3:** Associations between plasma phosphate and all-cause mortality in individuals with *versus* without type 2 diabetes

	Type 2 diabetes				No diabetes			
	All-cause mortality (^n^events/^n^total = 139/1790)				All-cause mortality (^n^events/^n^total = 1126/55380)			
	Low	Intermediate	High	*p*	Low	Intermediate	High	*p*
	0.34–0.85 mmol/L	0.85–0.98 mmol/L	0.99–1.52 mmol/L		0.24–0.85 mmol/L	0.85–0.98 mmol/L	0.99–1.83 mmol/L	
Person years	5672	5516	6471		183,920	171,347	177,475	
Events	45	37	57		418	338	370	
Crude incident rate per 1000 person-years	7.94	6.71	8.81		2.27	1.97	2.08	
Model 1	1.15 (0.74–1.78)	1.0 (ref)	**1.75 (1.14**–**2.67)**	**0.01**	0.96 (0.83–1.11)	1.0 (ref)	1.16 (1.00–1.36)	0.05
Model 2	1.18 (0.75–1.83)	1.0 (ref)	**1.69 (1.10**–**2.59)**	**0.02**	0.96 (0.83–1.12)	1.0 (ref)	1.12 (0.96–1.31)	0.14
Model 3	1.14 (0.73–1.79)	1.0 (ref)	**1.65 (1.06**–**2.56)**	**0.03**	0.96 (0.83–1.11)	1.0 (ref)	1.13 (0.97–1.31)	0.13
Model 4	1.07 (0.67–1.68)	1.0 (ref)	**1.73 (1.10–2.72)**	**0.02**	0.95 (0.82–1.10)	1.0 (ref)	1.12 (0.96–1.31)	0.14

Data are presented as hazard ratio (HR) plus 95% CI according to tertiles of plasma phosphorus

P values of < 0.05 were considered as clinical significant and are presented in bold

Model 1: adjusted for age and gender

Model 2: adjusted for Model 1 plus smoking, use of alcohol, BMI (categorical), HbA1c, and SBP

Model 3: adjusted for Model 2 plus LDL, eGFR, (corrected) plasma calcium, use of lipid lowering drugs, use of anti-thrombotic agents, use of anti-diabetic agents, use of diuretics, and use of vitamin D supplementation

Model 4: adjusted for Model 3 plus LLDS, education level, income level, marital stage, exercise and presence of comorbidities (COPD, cancer, dementia, epilepsy, liver cirrhosis, history of CVD)

*BMI* body mass index, *HbA1c* glycated haemoglobin, *SBP* systolic blood pressure, *LDL* low-density lipoprotein, *CVD* cardiovascular disease, *eGFR* estimated glomerular filtration rate, *LLDS* Lifelines Diet score, *COPD* chronic obstructive pulmonary disease, *CVD* cardiovascular disease

### Sensitivity analyses

Since the association between plasma phosphate and mortality could be driven by differences in kidney function, we performed a separate sensitivity analysis in which we excluded all individuals with an eGFR < 60 mL/min/1.73 m^2^. After exclusion of 91 individuals, the association between plasma phosphate and mortality remained statistically significant (Table 4).

**Table 4:** Associations between plasma phosphate and all-cause mortality in individuals with type 2 diabetes after exclusion of individuals with eGFR < 60 mL/min/1.73 m^2^

	Type 2 diabetes without CKD All-cause mortality (nevents/ntotal = 117/1595)			
	Low (0.35–0.85 mmol/L)	Intermediate (0.85–0.98 mmol/L)	High (0.99–1.52 mmol/L)	*p*
Person years	6121	5292	5656	
Events	38	30	49	
Crude incident rate per 1000 person-years	6.21	5.67	8.66	
Model 1	1.11 (0.70–1.84)	1.0 (ref)	**1.90 (1.18–2.99)**	**0.01**
Model 2	1.16 (0.71–1.88)	1.0 (ref)	**1.83 (1.15–2.93)**	**0.01**
Model 3	1.10 (0.67–1.79)	1.0 (ref)	**1.77 (1.10–2.85)**	**0.02**
Model 4	1.07 (0.64–1.77)	1.0 (ref)	**1.83 (1.12–2.99)**	**0.02**

Data are presented as hazard ratio (HR) plus 95% CI according to tertiles of plasma phosphorus

P values of < 0.05 were considered as clinical significant and are presented in bold

Model 1: adjusted for age and gender

Model 2: adjusted for Model 1 plus smoking, use of alcohol, BMI (categorical), HbA1c, and SBP

Model 3: adjusted for Model 2 plus LDL, eGFR, (corrected) plasma calcium, use of lipid lowering drugs, use of anti-thrombotic agents, use of anti-diabetic agents, use of diuretics, and use of vitamin D supplementation

Model 4: adjusted for Model 3 plus LLDS, education level, income level, marital stage, exercise and presence of comorbidities (COPD, cancer, dementia, epilepsy, liver cirrhosis, history of CVD)

*BMI* body mass index, *HbA1c* glycated haemoglobin, *SBP* systolic blood pressure, *LDL* low-density lipoprotein, *CVD* cardiovascular disease, *eGFR* estimated glomerular filtration rate, *LLDS* Lifelines Diet score, *COPD* chronic obstructive pulmonary disease, *CVD* cardiovascular disease

In a second sensitivity analysis, we excluded individuals with plasma phosphate outside the normal range (0.70–1.50 mmol/L). After exclusion of 152 individuals (151 with plasma phosphate < 0.70 mmol/L and one with plasma phosphate > 1.50 mmol/L), the results were highly similar (Additional file 1: Table S4).

To minimize the risk that (sub) clinical morbidity associated with phosphate levels could influence the outcome, we excluded individuals that had died within the first five years of follow-up in a third sensitivity analysis. After exclusion of 50 individuals, results remained statistically significant (full model HR 1.71 [95% CI 1.00–2.90]).

## Discussion

In this large population-based cohort, we found that higher plasma phosphate levels were significantly associated with a higher all-cause mortality risk, independent of potential confounders. Interestingly, we observed significant effect modification by type 2 diabetes, and found a stronger association between plasma phosphate and mortality in individuals with type 2 diabetes at baseline, compared with those free from diabetes. Results were consistent upon several sensitivity analyses. Thus, we interpret our findings as an indication that individuals with type 2 diabetes may be more susceptible to higher phosphate levels.

Although this study is the first to compare the association between plasma phosphate and mortality in individuals with and without diabetes, the results in our type 2 diabetes subpopulation are in line with previous studies in type 2 diabetes populations with and without chronic kidney disease (CKD) [21, 22]. Still, these studies reported much higher average phosphate levels than in the current study (1.16–1.43 mmol/L versus 0.93 mmol/L on average, respectively). While our study was based on a large population-based cohort, the aforementioned studies selected individuals from an outpatient hospital setting with also higher glucose levels and worse kidney function, which could explain these differences. The association between plasma phosphate and mortality has been reported previously in the general population, although effect modification by diabetes has not been previously described [15]. This is the first study to elucidate that even with plasma phosphate levels comparable to individuals without type 2 diabetes (0.93 vs 0.91 mmol/L), the association with mortality is stronger in individuals with type 2 diabetes. We also found a non-significant trend towards a higher mortality risk in participants with a lower phosphate level and with diabetes, while a lower phosphate level was associated with a lower mortality risk in those without diabetes (Fig. 2). Both a linear and a U-shaped relationship between phosphate and mortality have been reported in population-based cohort studies [20, 23], while previous (much smaller) studies in individuals with diabetes reported a linear association [21, 22]. The U-shaped association in our study might be explained by an increased susceptibility to mitochondrial dysfunction as a result of low ATP levels in patients with diabetes and hypophosphatemia [24].

Interestingly, women were overrepresented in the highest phosphate tertile in our study. Previous studies consistently report that postmenopausal woman have higher phosphate levels, compared to age-matched men, and suggested that declining estrogen levels after menopause causes sex differences in calcium and phosphate levels [25]. Adjustment for gender did not materially change the results, and we also found no effect modification by gender. In addition, we observed a non-significant trend suggesting that also low phosphate levels were associated with an increased mortality risk in individuals with type 2 diabetes. There are no comparative studies investigating these range of low phosphate levels in relation to mortality in type 2 diabetes. However, low phosphate levels can affect organ systems by reducing ATP synthesis resulting in e.g., acute heart failure, which may explain these results [26].

Because of the observational study design, we cannot draw firm conclusions about underlying mechanisms. However, we can speculate on potential pathways involved. Individuals with type 2 diabetes may be relatively susceptible for higher plasma phosphate levels, as hyperglycemia induces phosphate uptake by upregulation of PiT-1 [13, 14]. As a result of inflammation and oxidative stress in type 2 diabetes, PiT-1 upregulation can be induced by the interation of ligands such as advanced glycosylation end products (AGEs) with transcription factor nuclear factor-KB (NFκB) [27, 28]. Also, higher levels of tumor necrosis factor (TNF)- α, as found in type 2 diabetes, may induce NFκB and bone morphogenetic protein-2, both involved in PiT-1 upregulation [29, 30]. PiT-1, in turn, induces vascular calcification by promoting VSMCs to transition to an osteochondrocytic phenotype in the presence of phosphate [12]. While the presence of vascular calcification is an independent predictor of cardiovascular mortality in prior studies [31], the Lifelines cohort does not have data on vascular calcification, cardiovascular events or cause-specific mortality to substantiate the hypothesis that the observed association between phosphate and mortality was driven by vascular calcification or cardiovascular events, although it is likely that a considerable proportion of all-cause mortality was driven by cardiovascular disease. Individuals with lower plasma phosphate levels had a lower LLDS, indicative of lower diet quality. At the same time, adjusting for the LLDS did not materially change the association between phosphate and mortality, suggesting that differences in diet quality did not importantly contribute to this association.

We performed sensitivity analyses to address whether the association between plasma phosphate and mortality was driven by an eGFR < 60 mL/min/1.73 m^2^, by phosphate levels outside the reference range (0.70–1.50 mmol/L), or by early mortality. Plasma phosphate levels increase in moderate to advanced chronic kidney disease (eGFR < 60 mL/min/1.73 m^2^), driven by markedly increased blood levels of phosphate-regulating hormones such as fibroblast growth factor 23 and parathyroid hormone [32]. Interestingly, our findings were consistent in analyses restricted to individuals without CKD, suggesting that the association between higher phosphate levels and mortality in individuals with type 2 diabetes is not determined by impaired renal excretion of phosphate. Results were also similar in analyses restricted to individuals with phosphate levels in the normal range, and after exclusion of individuals who died within the first five years of follow-up.

A major strength of this study is the large population size, well-characterized cohort with data regarding type 2 diabetes status, medication use, and clinically relevant outcomes. Nevertheless, certain limitations of this study should be noted. First, the prevalence of type 2 diabetes in the Lifelines cohort (3%) is much lower than in the general Dutch population (5.8%) [33]. This may be due to selection bias towards healthy individuals and may limit the external validity of our cohort, but this does not necessarily impact the association between phosphate and mortality. Second, some patient-reported data are prone to recall bias, although this does not apply to the main exposure (phosphate) and outcome (all-cause mortality). Third, since phosphate was only measured at baseline, we could not take changes over time into account. Fourth, it was not possible to adjust for all conditions that may influence plasma phosphate, e.g., the presence of hypoparathyroidism. Thus, despite extensive adjustment in multivariable models, we cannot exclude residual confounding. Finally, no data on vascular calcification, cardiovascular events, or cause-specific mortality were available to confirm that the association with all-cause mortality was driven by cardiovascular disease.

In conclusion, we found that the association between higher plasma phosphate levels, even within the normal range, and mortality is stronger in individuals with type 2 diabetes than those without. Our findings set the stage for prospective studies investigating the impact of reducing high-normal plasma phosphate on health outcomes in individuals with type 2 diabetes. Furthermore, the association between low phosphate levels and mortality in diabetes deserves further investigation.

## Supplementary Information

**Additional file 1: Table S1.** Baseline characteristics of individuals with and without available laboratory data at baseline in the Lifelines cohort. **Table S2.** Baseline characteristics of individuals in the Lifelines cohort that had died during follow up. **Table S3.** Baseline characteristics of individuals with type 2 diabetes according to groups of plasma phosphate in the Lifelines cohort. **Table S4.** Associations between plasma phosphate and all-cause mortality in type 2 diabetes with exclusion of individuals with plasma phosphate outside the reference range.

## Data Availability

The datasets analysed during the current study are not publicly available because they contain information that could compromise the privacy of research participants.
