# Supplementary material for: Plasma phosphate and all-cause mortality in individuals with and without type 2 diabetes: the Dutch population-based lifelines cohort study
Source: Cardiovasc Diabetol. 2022 Apr 27;21:61. doi: 10.1186/s12933-022-01499-4 (PMC9047280; doi:10.1186/s12933-022-01499-4)
Supplement: Supplementary file 1 — Additional file 1: Table S1. Baseline characteristics of individuals with and without available laboratory data at baseline in the Lifelines cohort. Table S2. Baseline characteristics of individuals in the Lifelines cohort that had died during follow up. Table S3. Baseline characteristics of individuals with type 2 diabetes according to groups of plasma phosphate in the Lifelines cohort. Table S4. Associations between plasma phosphate and all-cause mortality in type 2 diabetes with exclusion of individuals with plasma phosphate outside the reference range. [file 12933_2022_1499_MOESM1_ESM.docx]

Additional file 1

Table S1. Baseline characteristics of individuals with and without available laboratory data at baseline in the Lifelines cohort

|  | **Individuals with available laboratory data**  **(*n* = 57 170)** | **Individuals without available laboratory data**  **(*n=*90 693)** |
| --- | --- | --- |
| Age (years) | 44 [36-51] | 45 [35-54] |
| Gender (male,%) | 23633(41) | 37971 (42) |
| Smoking (yes,%) | 11054 (20) | 18360 (20) |
| BMI (kg/m^2^) | 26.1 ± 4.3 | 26.1 ± 4.3 |
| Systolic blood pressure (mmHg) | 125 ± 15 | 126 ± 15 |
| Diastolic blood pressure(mmHg) | 74 ± 9 | 73 ± 9 |
| Died during follow up (%) | 1265 (2) | 1872 (2) |
| History of cardiovascular disease (%) | 1519 (3) | 2577 (3) |

Values are means ± standard deviation, medians (interquartile range) or proportions (%). P values of <0.05 were considered as clinical significant. *BMI* body mass index.

Table S2. Baseline characteristics of individuals in the Lifelines cohort that had died during follow up

|  | **Total (*n* =1265)** | **Type 2 diabetes (*n* =139)** | **Non diabetes (*n=*1126)** | **P value** |
| --- | --- | --- | --- | --- |
| **Demographics** |  |  |  |  |
| Age (years) | 62 [49-71] | 67 [60-75] | 61 [49-70] | **<0.001** |
| Gender (male,%) | 695 (55) | 90 (65) | 605 (54) | **0.01** |
| Smoking (yes,%) | 293 (23) | 27 (19) | 266 (24) | 0.23 |
| BMI (kg/m^2^) | 27.2 ± 4.4 | 29.7 ± 5.4 | 26.9 ± 4.2 | **<0.001** |
| History of cardiovascular disease (%) | 177 (14) | 48 (35) | 129 (12) | **<0.001** |
| Follow up period (months) | 70 (31) | 68 (30) | 70 (31) | 0.54 |
| eGFR (ml/min/1,73 m2) | 84 ± 18 | 80 ± 20 | 84 ± 17 | **0.003** |
| Phosphate (mmol/L) | 0.91 ± 0.17 | 0.94 ± 0.19 | 0.91 ± 0.17 | 0.12 |
| HbA1c (mmol/mol) | 41 ± 7 | 53 ± 10 | 39 ± 4 | **<0.001** |
| HbA1c (%) | 5.9 ± 1.0 | 7.0 ± 1.3 | 5.7 ± 0.6 | **<0.001** |
| HDL cholesterol (mmol/L) | 1.43 ± 0.42 | 1.22 ± 0.37 | 1.45 ± 0.42 | **<0.001** |

Values are means ± standard deviation, medians (interquartile range) or proportions (%). *BMI* body mass index, *eGFR* estimated glomerular filtration rate, *HbA1c* glycated haemoglobin, *HDL* high-density lipoprotein.

Table S3. Baseline characteristics of individuals with type 2 diabetes according to groups of plasma phosphate in the Lifelines cohort

|  |  | **Group 1 (*n* = 575)** | **Group 2 (*n* = 562)** | **Group 3 (*n* = 653)** |  |
| --- | --- | --- | --- | --- | --- |
|  | **Total (*n* =1 790)** |  |  |  |  |
|  |  | **Plasma phosphate 0.34 – 0.84 mmol/L** | **Plasma phosphate 0.85 – 0.99 mmol/L** | **Plasma phosphate 1.00– 1.82 mmol/L** | **P value** |
| **Demographics** |  |  |  |  |  |
| Age (years) | 57 [48-65] | 55 [47-65] | 58 [47-67] | 57 [48–65**]** | 0.22 |
| Gender (male,%) | 842 (47) | 406 (71) | 254 (45) | 182 (28) | **<0.001** |
| Smoking |  |  |  |  |  |
| No smoking (%) | 607 (34) | 184 (33) | 197 (36) | 226 (36) | 0.10 |
| Former smoker(%) | 813 (45) | 293(52) | 248 (45) | 272 (43) |  |
| Current smoker (%) | 324 (18) | 87 (15) | 104 (19) | 133 (21) |  |
| Alcohol use (units/week) | 7 [0-14] | 7 [0-14] | 7 [0-14] | 0 [0-14] |  |
| BMI (kg/m^2^) | 30.3 ± 5.6 | 30.4 ± 5.1 | 30.6 ± 5.7 | 30.1 ± 5.9 | 0.16 |
| Lifelines Diet Score | 25.0 ± 5.9 | 24.2 ± 6.1 | 24.9 ± 5.8 | 25.7 ± 5.9 | **0.003** |
| Systolic blood pressure (mmHg) | 135 ± 17 | 137 ± 15 | 136 ± 18 | 132 ± 17 | **<0.001** |
| Diastolic blood pressure(mmHg) | 76 ± 9 | 78 ± 9 | 76 ± 9 | 74 ± 9 | **<0.001** |
| History of cardiovascular disease (%) | 239 (13) | 77 (13) | 70 (13) | 92 (14) | 0.99 |
| Diabetes duration (years) | 5 [2-10] | 4 [2-8] | 5 [2-10] | 5 [2-10] | 0.32 |
| Other comorbidities (COPD, cancer, dementia, epilepsy, liver cirrhosis) (%) | 102 (6) | 34 (6) | 28 (5) | 40 (6) | 0.35 |
|  |  |  |  |  |  |
| Sodium (mmol/L) | 141 ± 2 | 141 ± 2 | 141 ± 2 | 141 ± 2 | 0.55 |
| Potassium (mmol/L) | 4.0 ± 0.3 | 4.0 ± 0.3 | 4.0 ± 0.3 | 4.0 ± 0.3 | 0.75 |
| Calcium (mmol/L) | 2.30 ± 0.09 | 2.29 ± 0.09 | 2.30 ± 0.09 | 2.31 ± 0.09 | **<0.001** |
| eGFR (ml/min/1,73 m2) | 89 ± 17 | 90 ± 16 | 89 ± 16 | 88 ± 17 | 0.45 |
| Phosphate (mmol/L) | 0.93 ± 0.17 | 0.76 ± 0.17 | 0.92 ± 0.17 | 1.08 ± 0.16 | **<0.001** |
| Glucose (mmol/L) | 7.4 ± 2.3 | 7.6 ± 2.4 | 7.4 ± 2.3 | 7.3 ± 2.2 | **0.008** |
| HbA1c (mmol/mol) | 52 ± 13 | 52 ± 14 | 52 ± 13 | 53 ± 13 | 0.68 |
| HbA1c(%) | 6.9 ± 1.7 | 6.9 ± 1.9 | 6.9 ± 1.7 | 6.9 ± 1.7 | 0.68 |
| HDL cholesterol (mmol/L) | 1.26 ± 0.36 | 1.19 ± 0.32 | 1.26 ± 0.35 | 1.32 ± 0.38 | **<0.001** |
| LDL cholesterol (mmol/L) | 2.94 ± 0.99 | 2.99 ± 0.99 | 2.93 ± 0.99 | 2.91 ± 1.00 | 0.35 |
| Triglycerides (mmol/L) | 1.44 [1.04-2.08] | 1.54 [1.08 – 2.22] | 1.39 [1.02 – 1.98] | 1.43 [1.02 – 2.06] | **0.005** |
| Total cholesterol (mmol/L) | 4.78 ± 1.13 | 4.81 ± 1.12 | 4.77 ± 1.14 | 4.77 ± 1.12 | 0.70 |
| Vitamin D supplement use (%) | 7 (0) | 1 (0) | 1 (0) | 5 (1) | 0.13 |
| Use of diuretics (%) | 333 (19) | 91 (16) | 111 (20) | 131 (20) | 0.12 |
| Use of lipid lowering drugs (%) | 823 (46) | 235 (41) | 281 (50) | 307 (47) | **0.005** |
| Use of anti-thrombotic agents (%) | 333 (19) | 111 (19) | 107 (19) | 115 (18) | 0.78 |
| Diabetes therapy |  |  |  |  |  |
| OBGLD (%) | 681 (38) | 216 (38) | 200 (36) | 265 (41) | 0.20 |
| Insulin (%) | 75 (4) | 20 (4) | 24 (4) | 31 (5) | 0.37 |
| Insulin and OBGLD (%) | 113 (6) | 19 (3) | 37 (7) | 57 (9) | **0.001** |

Values are means ± standard deviation, medians (interquartile range) or proportions (%). P values of <0.05 were considered as clinical significant. *BMI* body mass index, *eGFR* estimated glomerular filtration rate, *HbA1c* glycated haemoglobin, *HDL* high-density lipoprotein, *LDL* low-density lipoprotein, *OBGLD* oral blood glucose lowering drugs *COPD* chronic obstructive pulmonary disease.

Table S4. Associations between plasma phosphate and all-cause mortality in type 2 diabetes with exclusion of individuals with plasma phosphate outside the reference range

|  |  | **Type 2 diabetes** |  |  |
| --- | --- | --- | --- | --- |
| **All-cause mortality (^n^events/^n^total = 126/1638)** | | | | |
|  | **Low**  0.70 – 0.84 mmol/L | **Intermediate**  0.85 – 0.98 mmol/L | **High**  0.99 – 1.50 mmol/L | *p* |
| **Person years** | 3,845 | 5,178 | 5,988 |  |
| **Events** | 32 | 37 | 57 |  |
| **Crude incident rate per 1000**  **person- years** | 8.32 | 7.15 | 9.52 |  |
| **Model 1** | 1.10 (0.68 – 1.77) | 1.0 (ref) | **1.73 (1.13– 2.64)** | **0.01** |
| **Model 2** | 1.09 (0.68 – 1.76) | 1.0 (ref) | **1.66 (1.08 – 2.54)** | **0.02** |
| **Model 3** | 1.11 (0.68 – 1.79) | 1.0 (ref) | **1.60 (1.03 – 2.48)** | **0.03** |
| **Model 4** | 1.11 (0.69 – 1.81) | 1.0 (ref) | **1.64 (1.06 – 2.56)** | **0.03** |

Data are presented as hazard ratio (HR) plus 95% CI according to tertiles of plasma phosphorus. *BMI* body mass index, *HbA1c* glycated haemoglobin, *SBP* systolic blood pressure, *LDL* low-density lipoprotein, *CVD* cardiovascular disease, *eGFR* estimated glomerular filtration rate *LLDS* Lifelines Diet score *COPD* chronic obstructive pulmonary disease *CVD* cardiovascular disease.

Model 1: adjusted for age and gender
Model 2: adjusted for Model 1 plus smoking, use of alcohol, BMI (categorical), HbA1c, and SBP
Model 3: adjusted for Model 2 plus LDL, eGFR, (corrected) plasma calcium, use of lipid lowering drugs, use of anti-thrombotic agents, use of anti-diabetic agents, use of diuretics, and use of vitamin D supplementation
Model 4: adjusted for Model 3 plus LLDS, education level, income level, marital stage, exercise and presence of comorbidities (COPD, cancer, dementia, epilepsy, liver cirrhosis, history of CVD)
